# Leaders Condition the Work Experience: A Test of a Job Resources-Demands Model Invariance in Two Countries

**DOI:** 10.1155/2023/1353289

**Published:** 2023-02-22

**Authors:** Rita Berger, Sharon Glazer, David Leiva

**Affiliations:** ^1^University of Barcelona, Barcelona, Spain; ^2^The University of Baltimore, Baltimore, USA

## Abstract

**Background:**

In today's healthcare sector, promoting health among employees is more relevant than ever. Health-promoting leadership styles, such as transformational leadership, can positively affect staff well-being, but research on laissez-faire leadership is particularly sparse, though it is believed to be detrimental. Past research suggests that leadership conditions work experiences and can exacerbate or mitigate role stressors that result in individual outcomes. *Method(s)*. Questionnaires were administered to nurses in the USA (*n* = 208) and Spain (*n* = 220), with a five- and eight-week separation, respectively.

**Results:**

Transformational leadership has a negative and laissez-faire leadership has a positive relationship with adverse outcomes. Furthermore, role overload and conflict mediate the relationship between leadership styles and outcomes. *Conclusion(s)*. The study provides incremental evidence of the negative implications of laissez-faire leadership compared with the positive implications of transformational leadership on outcomes via role stressors as motivational mechanisms. *Implications for Nursing Management*. Learning about the medium-term implications of leadership styles on stressors and health-related outcomes would enrich opportunities for leadership training in organizations.

## 1. Background

According to Katz and Kahn's [1] social environment model, the work context influences the psychological experiences of stressors. Leaders are a pivotal component of the work context [2] and are the first in line to promote healthy work practices and worker well-being [3]. Extant literature on leadership has mainly examined the relationship between leadership and performance-related outcomes, and less often leadership in relation to well-being [3]; a few studies show the effects of leadership on subordinate well-being, including anxiety, burnout, stress, turnover [4–6], and mental health [7] as first-level outcomes that mediate the relationship between leadership style and performance. However, few studies have analyzed mediating factors [3, 4, 8] as mechanisms for the relationship between leadership style and well-being.

**Figure 1:**
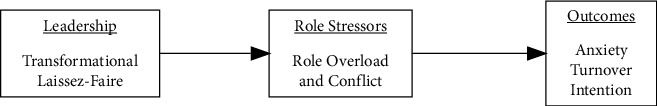
Proposed a job resources-demands model.

The Job Demands-Resources (JD-R) model [9] positions leadership as a key variable influencing perceptions of role demands and resources (e.g., [10]). Thus, positive behavioral qualities in leadership are viewed as a resource to mitigate the adverse effects of stressors [4, 5, 10]. However, health-promoting leadership, such as transformational leadership, can also prevent subordinates' experienced stressors, and, therefore, it also serves as an antecedent to job demands. Therefore, we propose and test a job resources-demands model (see Figure 1).

Results from studies in different countries in the healthcare sector show that transformational leaders (i.e., leaders that inspire, give individualized consideration, intellectually stimulate, and idealize influence) [11] help prevent job demands that affect well-being (e.g., [5, 12, 13]), whereas laissez-faire leadership (i.e., leaders that are unengaged with their subordinates) was not predictive of subordinate anxiety [14]. Transformational leadership is considered a positive resource, as such leaders focus on employee growth and development; however, laissez-faire leaders reflect negative behavioral qualities in leadership, as they neither care about the person nor the tasks. Laissez-faire leaders are unlikely to prevent stressors and strains. We contend that leaders condition subordinates' experiences, including stressors they perceive, which in turn relate to well-being outcomes.

As leaders influence subordinates' experiences in the workplace [15], it should only make sense to examine the favorably resourced context as an antecedent to perceptions of stressors and well-being. We, therefore, propose, per our JR-D model, that employees' experiences in a health-promoting (transformational) leadership context prevent stressors that lead to adverse outcomes, whereas reports of a laissez-faire leader will intensify them. Moreover, stressors' implications on strains will be weaker because of the positive behavioral qualities of the transformational leader, but stronger when faced with a laissez-faire leader.

The focal outcomes in this study are anxiety and the intention to leave the organization (aka. turnover intention). Anxiety is a psychophysiological response that can manifest as tightness in the chest, fear or worry, and even panic attacks [16]. It is an important first indication of psychological distress [17]. Turnover intention (hereto turnover) reflects a person's behavioral inclination to leave his or her workplace [16]. Findings associated with turnover can help make a stronger argument to management about why they might invest in leadership development. Current results show that transformational leadership indirectly and negatively relates to turnover via work stress [6] and irritation via job demands [5]. However, research on laissez-faire leadership, a passive leadership style, is scarce. In one study, laissez-faire leadership had no effect on anxiety [14], but in another, passive-avoidant leadership resulted in anxiety via role ambiguity [18]. Therefore, we expect leadership styles to indirectly relate to anxiety and turnover via role stressors.

### 1.1. Current Study

This study uses a two-wave approach to study how transformational and laissez-faire leadership relates to subordinates' perceptions of role overload and conflict (ROC), which further impact subordinates' reports of anxiety (a personal consequence) and turnover intention (an organizational consequence). The decision to examine leadership styles in relation to role stressors and strains is consistent with the theoretical linkage of stressors as the motivational mechanism that could explain the implications of leadership styles on well-being outcomes [3]. Moreover, theoretically, a leader's behaviors have both long-term and short-term impacts (e.g., [19]. In the short term, it should immediately relate to the experience of stressors, and in the long term, it should affect outcomes via stressors. Therefore, we assessed leadership and stressors at Time 1 (T1) and outcomes at Time 2 (T2).

Our study presents two novel contributions. First, consistent with the results of an earlier study [18], we are here proposing a job resources-demands (JR-D, instead of JD-R) model to analyze work role stressors as job demands that mediate the relationship between both active and inactive leadership styles and well-being-related outcomes. This point is particularly relevant given the scarcity of research linking inactive (laissez-faire) leadership with well-being via the motivational mechanisms of role stressors. Second, we demonstrate model invariance in a two-country study. Our samples came from the same profession, but from different countries. Burgeoning research shows that although the implications of a transformational leadership style may be positive across cultures, the relationship is stronger in high uncertainty avoidant cultures [20]. Watts et al. [20] identified Spain, a Latin European country, as high on uncertainty avoidance, and the USA as low on uncertainty avoidance. Thus, the second aim is to show consistency in relationship patterns or to give credence to Watts et al.'s findings.

### 1.2. Hypotheses (H)

Regardless of cultural context,

  H_1_: laissez-faire leadership (Time 1 or T1) will positively relate to (a) anxiety and (b) turnover intention (Time 2 or T2)
  H_2_: role overload and conflict (T1) will mediate the effect of laissez-faire leadership on (a) anxiety and (b) turnover intention (T2)
  H_3_: transformational leadership (T1) will negatively relate to anxiety and turnover intentions (T2)
  H_4_: role overload and conflict (T1) will mediate the relationship between transformational leadership (T1) and both (a) anxiety and (b) turnover intention (at T2)

## 2. Methods

### 2.1. Participants

Participants included 428 nurses working in two nursing homes in Spain and one hospital in the USA. These countries were chosen because of their clear cultural values differences in several cultural values, chief among them, uncertainty avoidance [21], which could influence the perception of leadership and role stressors [22], as could their differing healthcare systems [23].

### 2.2. Measures

With the exception of the questions on laissez-faire leadership, all other survey items were rated on a 7-pointLikert-type scale from 1 = “strongly disagree” to 7 = “strongly agree” and translated into Spanish following the guidelines of the International Test Commission [24].

#### 2.2.1. Leadership Style

Eight items come from the Human Systems Audit transformational leadership short scale [25]. An example item is “S/he promotes the use of intelligence to overcome obstacles.” Another four items addressing laissez-faire leadership were adopted from the MLQ-5X Multifactor Leadership Questionnaire (MLQ) 5X [26, 27]. An example of a laissez-faire leadership item is “Is absent when needed.” Leadership items were rated on a 5-point scale, from 0, “never,” to 4, “almost always.” In Spain and the USA, Cronbach alphas for transformational leadership were 0.97 and 0.98, respectively, and for laissez-faire leadership, alphas were 0.73 and 0.76, respectively.

#### 2.2.2. Role Overload and Conflict (ROC)

Consistent with Katz and Kahn [1], a single index of ROC was created. Four role overload and three role conflict items were drawn from a previously cross-culturally validated measure of role stressors [16] (e.g., “It seems like I have too much work for one person to do,” and “I receive incompatible requests from two or more people”). Cronbach alpha coefficients were modest in the Spanish sample (*α* = 0.63), but acceptable in the U.S. sample (*α* = 0.83, see main diagonals of Table 1). Low alpha coefficients are not unusual when a measure developed in one language is administered (after proper translation and back translation) into another language, particularly Spanish [28].

#### 2.2.3. Outcomes

We drew on four items (e.g., “I have felt fidgety or nervous as a result of my job”) from Parker and Decotiis [29] to assess anxiety. Cronbach alpha coefficients were strong in both Spain (*α* = 0.85) and the USA (*α* = 0.91). Furthermore, to assess turnover intention, we adopted three items (e.g., “I often think about quitting”) from Seashore et al. [30]. Reliability coefficients were strong in Spain (*α* = 0.87) and the USA (*α* = 0.88).

### 2.3. Procedures

The first and second authors secured agreements for data collection at the data collection sites and received approval for data collection from the second author's Institutional Review Board. Paper-pencil surveys were administered to nurses in each healthcare institution. The surveys included an informed consent form. Respondents were under no obligation to complete the survey, which was both entirely voluntary and anonymous. In the USA, an envelope addressed to the second author was appended to the survey; completed surveys were sent internally to a specific office for retrieval. Nurses in Spain completed surveys during specified work hours, sealed them in envelopes, and returned them to the hospital research director, who presided over the survey distribution. Data were collected at two times (T1 and T2), separated by five (USA) and eight (Spain) weeks (during the data collection times permitted by the administrative staff of the facilities). T1 surveyed stressors, strains, and leadership styles. T2 surveyed stressors and strains. As the surveys were anonymous, all nurses received both surveys. A self-generated identification code included on both surveys, along with some key demographics (i.e., age and sex), was used to match respondents across both surveys. Nurses who completed T1, but not T2 or T2, but not T1 are not included in the study's analyses.

### 2.4. Data Analysis

Descriptive analyses and structural equation modeling were used to test hypotheses. Statistical analyses were conducted using R software version 3.4.4 [31]. Additionally, the psych package [32] was employed for some of the psychometric analyses, whereas the lavaan Package [33] was used for carrying out the Structural Equation Modeling (SEM). SEM models were estimated using full information maximum likelihood (FIML) and robust standard errors (Huber-White). Although there were missing data, we assumed that the missing data were completely at random (MCAR) since statistical tests yielded nonsignificant results in the U.S. sample (*p* = 0.66) and, thus, the chosen estimation procedure can be regarded as adequate. Concerning missing data distributions along the two measurement times, 29.8% of participants presented missing scores in the US sample (distributions among scales: *M* = 17.07%, SD = 18.03%) at T1, whilst no missing data patterns were found at the Spanish sample. As for the second measurement wave, 37.50% of responses had missing data in the US sample (scales distributions: *M* = 20.43%, SD = 22.78%) and 24.09% in the Spanish sample (*M* = 24.09%, SD = 0.0%)).

Measurement and structural models were estimated following a two-step approach in order to avoid problems that might arise when interpreting both models simultaneously. In total, we estimated the following three SEM nested models: a first model (Model 1) that takes into account the effect of leadership style over the outcome variables (either turnover intention or anxiety); a second model (Model 2) in which ROC fully mediates the effect of leadership over the outcome variables; a third model(Model 3) implies the partial mediation of ROC in the relationship between leadership and the outcomes, that is to say, direct and indirect effects were included in the model. As for the interpretation of the goodness of fit related to these models, we employed comparative fit and Tucker Lewis indices (CFI and TLI, respectively; values over 0.90 correspond to an acceptable fit in both cases), root mean square error of approximation as well as its 90% confidence interval (RMSEA; RMSEA <0.05 is considered adequate enough, whereas regarding the CI, lower bounds lower than 0.05, ideally closer to 0.00, and upper bounds lower than 0.08 would imply an acceptable fit [34]. To compare the nested models, we also obtained the sample-size adjusted Bayesian Information Criterion (saBIC; in general, a lower value implies a better fit). Bias-corrected and accelerated confidence intervals were obtained by means of a bootstrapping procedure (5000 samples generated within each run) for estimating the indirect effects. Since several authors (cf., [35] discouraged the use of asymptotic theory when testing indirect effects (i.e., tests assuming normality for the sampling distribution of the test statistic), we assumed a significant indirect effect whenever the confidence interval did not include zero.

## 3. Results

The samples consisted primarily of female nurses (91.8% in Spain and 96.1% in the USA). Nurses' ages ranged from 19 to 65 (*M* = 43.3 and SD = 11.4) years in Spain and 26 to 66 (*M* = 47.5 and SD = 10.29) years in the USA. In both countries, most of the nurses worked full-time (85.9% in Spain and 70.6% in the USA). Tenure ranged from brand new to 32 (*M* = 7.01 and SD = 6.75) years in Spain, and from brand new to 46 (*M* = 10.95 and SD = 10.2) years in the USA.

Per Table 1, Hypotheses 1 (H_1_) and 3 (H_3_) were supported in the overall and Spanish samples. In the Spanish sample, laissez-faire leadership (H_1_) at T1 positively related with both turnover intention (*r* = 0.21, *p*  <  0.01) and anxiety (*r* = 0.23, *p*  <  0.01), whereas transformational leadership style (H_3_) at T1 negatively related with turnover intention (*r* = −0.29, *p*  <  0.01) and anxiety (*r* = −0.31, *p*  <  0.01) at T2. In contrast, laissez-faire leadership style negatively correlated with anxiety (*r* = −0.32, *p*  <  0.05) in the U.S. sample, but no significant correlations were obtained between transformational leadership style and turnover intention measured at T2. All correlations differed significantly from zero when analyzing the two samples combined (i.e., the overall sample).

### 3.1. Role Overload and Conflict as Mediator of Laissez-Faire Leadership and Strains

#### 3.1.1. Overall Sample

**Figure 2:**
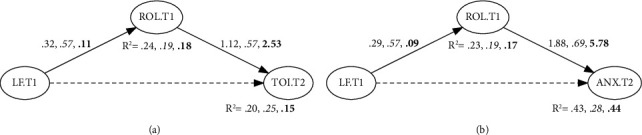
Path diagram of estimated coefficients in the overall, American (italic), and Spanish samples (bold), taking into account the full mediation model (model 2) for the laissez-faire effect over perceived strains. *Note*. LF.T1 = Laissez-faire scores at time 1 (T1); ROL.T1 = role overload and conflict at T1; TOI.T2 = turnover intention at time 2 (T2); anxiety.T2 = anxiety scores at T2.

Table 2 summarizes all the SEM models, including the laissez-faire scores as the main predictor for the samples. The models for the overall sample concerning turnover intention, showed acceptable fit as both CFI and LTI were greater than 0.90 and RMSEA values were lower than 0.08. The partial and full mediation models were comparatively better than the model including the direct effect of leadership over turnover intention (see Figure 2). Given the absence of statistically significant differences between the previous two models (Δ*χ*^2^ (1) = 1.45; *p*=0.23), we opted for the most parsimonious one (Model 2). The indirect effect of laissez-faire leadership on turnover intention, mediated by ROC, can be considered significant given the obtained confidence interval (estimate = 0.359; 95% CI = 0.21,0.509).

The SEM models related to anxiety fit the data adequately (CFI and LTI values over 0.90 and RMSEA below 0.08), except for the direct effect model. The full mediation model could be considered the better model according to saBIC indices and a LR test comparing the abovementioned model with the partial mediation model (Δ*χ*^2^ (1) = 0.00; *p*=0.98). The estimated indirect effect of laissez-faire leadership on anxiety at T2 was 0.55, and its confidence interval did not include the zero value (95% CI = 0.36, 0.74). According to these findings, we found support for H_2_ in the combined sample; the amount of variance accounted for in the outcome variables via the mediation models was 20% for turnover intention and 43% for anxiety.

#### 3.1.2. U.S. Sample

Table 2 shows the results for the SEM models in the U.S. sample including laissez-faire leadership as the predictor variable (see Table 2 (b)). A poor fit to the data can be assumed in all models (CFI and LTI values below 0.95 and RMSEA over 0.08). As found previously, we assumed the equivalence of the full mediation model to the partial mediation one in terms of goodness of fit (Δ*χ*^2^(1) = 0.24; *p*=0.62). The indirect effect of laissez-faire leadership on turnover intention, mediated by ROC, can be considered significant given the obtained confidence interval (estimate = 0.32; 95% CI = 0.11, 0.54).

Concerning anxiety, the three SEM models showed a poor fit to the data by examining the goodness of fit indices used in the current study. As it occurred in other cases, the full mediation model could be considered the better model after comparing saBIC indices and carrying out a LR test with a partial mediation model (Δ*χ*^2^(1) = 0.34; *p*=0.56). The estimated indirect effect of laissez-faire leadership on anxiety at T2 was 0.39, and its confidence interval did not include the zero value (95% CI = 0.14, 0.65). Thus, H_2_ was supported in the U.S. sample when taking laissez-faire leadership as the predictor for both response variables. In this regard, full mediation models achieved *R*-squared indices of 0.25 and 0.28 for turnover intention and anxiety at T2, respectively.

#### 3.1.3. Spanish Sample

Focusing on turnover intention, the models showed a good fit, as both CFI and LTI were greater than 0.95 and RMSEA values were lower than 0.05. Even the Chi-square test indicated a good fit to the data in Models 2 and 3. The partial and full mediation models were comparatively better than the remaining ones looking at the saBIC indices. Given the absence of statistically significant differences between the previous two models (Δ*χ*^2^(1) = 1.23; *p*=0.27), we opted for the most parsimonious one (Model 2). The estimated indirect effect showed a positive effect of laissez-faire leadership in relation to turnover intention as mediated by ROC (estimate = 0.289; 95% CI = 0.09, 0.49).

When examining anxiety as an outcome, we found that the best models in terms of the combined criteria seen so far were the mediation models (Models 2 and 3). For Model 2, since the likelihood ratio test did not yield significant differences between these models (Δ*χ*^2^(1) = 0.01; *p*=0.93), we kept Model 2 as the most fitted one. The indirect effect of laissez-faire leadership on anxiety mediated by ROC was 0.52 (95% CI = 0.23, 0.81). Thus, when laissez-faire leadership was accounted for as the predictor, H_2_ was supported. Full mediation models achieved *R*-squared indices of 0.15 and 0.44 for turnover intention and anxiety at T2, respectively.

### 3.2. Role Overload and Conflict as Mediator of Transformational Leadership and Strains

#### 3.2.1. Overall Sample

**Figure 3:**
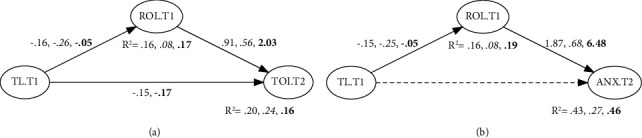
Path diagram of estimated coefficients in the overall, American (italic), and Spanish samples (bold), taking into account the full (model 2) or the partial mediation (model 3) model for the transformational leadership effect over perceived strains. *Note*. TL.T1 = transformational leadership scores at time 1 (T1); ROL.T1 = role overload and conflict at T1; TOI.T2 = turnover intention at time 2 (T2); anxiety.T2 = anxiety scores at T2.

Table 3 (a) shows results for the SEM models including transformational leadership in the two samples. If the turnover intention is included as the response, an acceptable fit can be assumed in the three models looking at the CFI and LTI (all values greater than 0.90) and the RMSEA (<0.08) except the direct effect model (RMSEA = 0.08). Full and partial mediation models seemed to be better than the alternative after inspecting the information criteria (see Figure 3). The partial mediation model is considered the best one since significant differences were found in terms of goodness of fit (Δ*χ*^2^(1) = 4.11; *p*=0.04). The indirect effects of transformational leadership on turnover intention mediated by ROC were estimated to be −0.14, and can be regarded as significant since its Bootstrap CI did not include zero (95% CI = −0.21, −0.075).

The SEM models fit the data reasonably well when including anxiety as the response (CFI and LTI values over 0.90 and RMSEA below 0.08), with the exception of model 1 (RMSEA = 0.08). The full mediation model can be preferred over the partial mediation alternative since the saBIC index of the first model is lower and the LR test yielded nonsignificant differences between the two models (Δ*χ*^2^(1) = 0.25; *p*=0.62). The estimated mediated effect of transformational leadership on anxiety at T2 was −0.28, and its confidence interval did not include the zero value (95% CI = −0.38, −0.18). Therefore, overall, we supported H_4_; the mediation models, including turnover intention and anxiety as outcomes at T2, achieved 20% and 43% of explained variance, respectively.

#### 3.2.2. U.S. Sample

Results for the SEM models including transformational leadership in the U.S. sample are shown in Table 3 (b). Acceptable fit can be assumed in the three models looking at the CFI and LTI (all values greater than 0.90), but not according to the RMSEA (>0.08). According to the saBIC indices, full and partial mediation models seemed to be better than the alternatives. The full mediation model is considered the best one once we assume both mediation models equivalent in terms of goodness of fit (Δ*χ*^2^(1) = 0.18; *p*=0.67). Transformational leadership's indirect effects on turnover intention mediated by ROC were estimated to be −0.14, and can be regarded as significant since its Bootstrap CI did not include the zero (95% CI = −0.26, −0.026).

Concerning anxiety, the three SEM models fit poorly to the data (most of the CFI and LTI values below 0.90 and RMSEA >0.08). The full mediation model could be considered better after comparing saBIC indices and carrying out a LR test with a partial mediation model (Δ*χ*^2^(1) = 0.16; *p*=0.69). The estimated mediated effect of transformational leadership over anxiety at T2 was −0.17, and its confidence interval did not include the zero value (95% CI = −0.22, −0.072). Thus, in the U.S. sample, we supported H_4_; the mediation models, including turnover intention and anxiety as outcomes at T2, achieved 19% and 27% of explained variance, respectively.

#### 3.2.3. Spanish Sample

The main results regarding SEM models, taking into account transformational leadership, are shown in Table 3 (c). In the three studied models, we included transformational leadership as the predictor, turnover intention or anxiety as outcomes, and ROC as the mediator. With turnover intention as the outcome, the three models did yield a reasonably good fit in terms of CFI and TLI (with values over 0.94), and RMSEA (values being <0.08, except for model 1 which is equal to 0.08). When comparing the models in terms of information criterion, Models 2 and 3 (i.e., full and partial mediation models) present better goodness of fit. Carrying out a likelihood ratio test, (Yuan–Bentler scaled LR test), we found significant differences between the models (Δ*χ*^2^(1) = 4.30; *p*=0.04) and thus advocated for the partial mediation model. By using the bootstrapping procedure, we obtained a confidence interval for the indirect effect which did not include the zero value (estimate = −0.12; 95% CI = −0.20, −0.033).

Concerning anxiety at T2, models 2 and 3 were similar in terms of goodness of fit (CFI = 0.94; LTI = 0.93; and RMSEA = 0.07) and better than the other model when comparing saBICs. Since no significant differences between them were found (Δ*χ*^2^(1) = 1.07; *p*=0.30), we identified the full mediation model as the best one. The mediated effect of transformational leadership on turnover intention through ROC was estimated to be −0.30 (95% CI = −0.44, −0.16). Thus, when accounting for transformational leadership as the predictor, H_4_ was supported. Full mediation models explained 16% and 46% of the variability in turnover intention and anxiety variables at T2, respectively.

## 4. Discussion

This study aimed to examine the role of a supervisor's leadership style as a factor that conditions a subordinate's work environment, which then stimulates an individual's perceived ROC and subsequent psychophysiological and behavioral intentional responses, namely, anxiety and turnover intention. Furthermore, these relationships were studied in two countries to explore their portability. However, the literature on this topic from different countries is too scant to be able to derive cross-cultural hypotheses, and therefore, cultural explanations are post hoc.

As expected in H_1_, nurses perceived that their supervisor employed a laissez-faire leadership style related to nurses' higher levels of anxiety and turnover intention, but only in the Spanish sample. Similarly, transformational leadership is negatively related to anxiety and turnover intention in the Spanish, but it is negatively related to turnover intention in the U.S. sample (H_3_). That there was no direct link between leadership style and strains for the U.S. sample was also found in Lyons and Schneider [36]. Specifically, transformational and transactional leadership styles had no effect on positive affect (a variable that can be considered the opposite of anxiety).

One might explain these results from a cultural lens. We surmise, post hoc, that there was no direct link between leadership style and anxiety due to the U.S. endorsement of Mastery values [37]. Mastery cultures expect that individuals are responsible for their own responses to environmental conditions. Therefore, U.S. participants in this study and Lyons and Schneider's [36] study may be influenced more by the culture-level values of Mastery, wherein it is expected that individuals “get…ahead through active self-assertion” [37], p. 28) and are, therefore, responsible for their own interpretation and responses to environmental conditions. Per Glazer et al. [38] Mastery cultures reinforce individual-level internal locus of control; in countries where the internal locus of control (i.e., a person believes that he or she is responsible for what happens to him or her) is stronger (e.g., the USA), those with an internal locus of control would, in fact, have less job stress, but in countries where the external locus of control (i.e., a person believes that others are responsible for what happens to him or her) is stronger, a person with an internal locus of control would, in fact, experience more job stress. U.S. leaders, therefore, may not be a direct influence on individuals' responses, but instead, the influence over strains may be driven by the culture's emphasis on Mastery and one's internal locus of control.

In contrast, Spanish culture strongly endorses egalitarian values (emphasizing others' welfare) and intellectual autonomy (i.e., the culture reinforces individual pursuit of independent ideas and intellectual stimulation) [37]. Leaders can either hinder or enable such pursuits, and therefore they may be viewed as directly responsible for employee well-being.

Per H_2_, laissez-faire leadership was positively related to ROC, which mediated the relationship between laissez-faire leadership and both anxiety and turnover intention. Skogstad et al. [39] also found a positive link between laissez-faire leadership and role stressors. Our study results suggest that laissez-faire leadership supports a noxious work environment that can lead to nurses' perceptions of ROC, increased anxiety, and greater turnover intention. Furthermore, supporting H_4_, transformational leadership is related to lower ROC, which is further related to lower anxiety and turnover intention. In other words, stressors (ROC) are a psychological process variable linking leadership style with psychological and behavioral outcomes [18]. Fernet et al. [12] also found that (emotional, cognitive, and physical) job demands mediated the relationship between transformational leadership and strain, specifically burnout, amongst a sample of French-Canadian nurses and school administrators, but as with the current study's results regarding U.S. study participants, transformational leadership did not directly relate to anxiety. This was also confirmed by Berger et al. [18] and Nielsen et al. [13]. Nielsen et al. found that work characteristics at T1 and T2 mediated the relationship between transformational leadership at T1 and well-being at T2, but there was no direct link between transformational leadership at T1 and well-being at T2. Thus, a transformational leader helps to reduce perceived ROC, which has an immediate effect on mitigating feelings of anxiety and turnover intention. In other words, transformational leadership stimulates a healthy work environment [36].

Despite the promising results, several limitations need to be addressed in future studies. First, the U.S. sample size is smaller than the Spanish one, and it is mainly due to attrition. A better sample size would be at least 200 nurses [40]. Nonetheless, SEM results demonstrate invariance between the two country samples. Second, Cronbach alpha reliability coefficients for the Spanish sample are low for the ROC measure. To improve upon this finding, we recommend retaining all 10 ROC survey items, per the original measure adapted from Glazer and Beehr [16]. These constructs were both valid and culturally invariant in their four-country study (albeit it did not include Spain). Third, this study focused on transformational and laissez-faire leadership only. The nurses' mean scores on laissez-faire leadership were quite low, particularly in Spain; thus, it is difficult to affirm the findings. Nonetheless, the findings are consistent with theory, so we do not dismiss them.

Given that our results were mostly invariant between the two countries, we have confidence that leaders condition subordinates' experiences of the work environment. Still, more cross-cultural research is needed on the effects of an inactive leadership style compared to other leadership styles on more worker attitudes, affects, and behaviors. Furthermore, a real-world intervention in which leaders are trained to adopt a transformational leadership would yield greater conclusive evidence that leaders condition experiences in the work environment.

## 5. Conclusions

The findings of this study demonstrate that leaders are a part of the work environment and condition work experiences. Specifically, leadership style relates to health and well-being through work stressors [12, 13]. Moreover, the study findings are being captured for the first time captured in a field study of healthcare providers in two countries. In both Spain and the USA, leadership style directly affected psychological processes (i.e., the perception of ROC) that then led one to experience anxiety or turnover intention. Theoretically, when it comes to leadership, we recommend revising the JD-R theory as the JR-D theory.

### 5.1. Implications for Nursing Management

Practically, management might take note that a healthy leadership style is one that emulates an engaged and considerate leader. These findings are important, as a nurse supervisor's leadership style has indirect implications on patient satisfaction with the quality of care [41]. Management should consider offering leadership training to ensure a more transformational style that would reduce anxiety and turnover and preserve quality healthcare.

## Figures and Tables

**Table 1:** Means and standard deviations, correlations (upper diagonal), sample sizes (lower diagonal), and Cronbach's alpha coefficients diagonal) for the overall (a), the U.S. (b), and the Spanish (c) samples.

	Time	*M*	SD	1	2	3	4	5
*(a) Overall sample*								
(1) Laissez-faire	1	1.91	0.96	(0.75)	−0.61^*∗∗*^	0.36^*∗∗*^	0.26^*∗∗*^	0.25^*∗∗*^
(2) Transformational	1	5.20	1.62	337	(0.97)	−0.33^*∗∗*^	−0.27^*∗∗*^	−0.29^*∗∗*^
(3) ROC	1	4.28	1.14	340	360	(0.72)	0.48^*∗∗*^	0.33^*∗∗*^
(4) Anxiety	2	3.26	1.64	286	298	302	(0.86)	0.49^*∗∗*^
(5) Turnover intention	2	2.25	1.54	286	298	302	352	(0.91)

*(b) US sample*								
(1) Laissez-faire	1	2.21	0.98	(0.76)	−0.66^*∗∗*^	0.45^*∗∗*^	0.32^*∗*^	0.28
(2) Transformational	1	4.89	1.67	117	(0.98)	−0.34^*∗∗*^	−0.12	−0.22
(3) ROC	1	4.36	1.25	120	140	(0.83)	0.52^*∗∗*^	0.40^*∗∗*^
(4) Anxiety	2	3.39	1.66	66	78	82	(0.91)	0.49^*∗∗*^
(5) Turnover intention	2	2.65	1.52	66	78	82	132	(0.88)

*(c) Spanish sample*								
(1) Laissez-faire	1	1.74	0.91	(0.73)	−0.57^*∗∗*^	0.29^*∗∗*^	0.23^*∗∗*^	0.21^*∗∗*^
(2) Transformational	1	5.40	1.57	220	(0.97)	−0.31^*∗∗*^	−0.31^*∗∗*^	−0.29^*∗∗*^
(3) ROC	1	4.23	1.05	220	220	(0.63)	0.47^*∗∗*^	0.29^*∗∗*^
(4) Anxiety	2	3.18	1.62	220	220	220	(0.85)	0.49^*∗∗*^
(5) Turnover intention	2	2.00	1.50	220	220	220	220	(0.92)

*Note.* ^*∗*^ *p*  <  0.05; ^*∗∗*^*p*  <  0.01. Laissez-faire and transformational refer to leadership styles. ROC = role overload and conflict.

**Table 2:** Results for the structural models studied using the laissez-faire leadership variable as the predictor in the overall (a), American (b), and Spanish samples (c). Role overload and conflict (mediator) were included in models 2 and 3.

	(a) Overall sample			(b) U.S. sample			(c) Spanish sample		
	*Response: Turnover Intention*			*Response: Turnover Intention*			*Response: Turnover Intention*		
	*Model 1*	*Model 2*	*Model 3*	*Model 1*	*Model 2*	*Model 3*	*Model 1*	*Model 2*	*Model 3*
Chi-square	176.72	157.76	156.13	176.30	166.92	166.59	104.35	93.73	92.47
*Df*	75	75	74	75	75	74	75	75	74
*p* value	<0.001	<0.001	<0.001	<0.001	<0.001	<0.001	0.01	0.07	0.07
CFI	0.94	0.95	0.95	0.88	0.89	0.89	0.97	0.98	0.98
TLI	0.92	0.94	0.94	0.85	0.86	0.86	0.96	0.97	0.97
RMSEA (90% CI)	0.06 (0.05–0.07)	0.05 (0.04–0.06)	0.05 (0.04–0.06)	0.08 (0.07–0.1)	0.08 (0.06–0.09)	0.08 (0.06–0.09)	0.04 (0.02–0.06)	0.03 (0.0–0.05)	0.03 (0.0–0.05)
SaBIC	17964.61	17945.66	1946.90	6873.04	6863.67	6865.47	10866.1	10855.48	10856.44

	*Response: Anxiety*			*Response: Anxiety*			*Response: Anxiety*		
	*Model 1*	*Model 2*	*Model 3*	*Model 1*	*Model 2*	*Model 3*	*Model 1*	*Model 2*	*Model 3*
Chi-square	284.04	218.66	218.66	227.92	216.14	215.80	187.96	140.14	140.13
*Df*	88	88	87	88	88	87	88	88	87
*p* value	<0.001	<0.001	<0.001	<0.001	<0.001	<0.001	<0.001	<0.001	<0.001
CFI	0.88	0.92	0.92	0.86	0.87	0.87	0.88	0.94	0.93
TLI	0.85	0.9	0.9	0.83	0.85	0.84	0.85	0.92	0.92
RMSEA (90% CI)	0.07 (0.06–0.08)	0.06 (0.05–0.07)	0.06 (0.05–0.07)	0.09 (0.07–0.1)	0.08 (0.07–0.1)	0.09 (0.07–0.1)	0.07 (0.06–0.09)	0.05 (0.04–0.07)	0.05 (0.04–0.07)
SaBIC	19857.64	19792.26	19795.13	7365.26	7353.48	7355.28	12207.64	12159.82	12162.03

*Note.* Model 1: direct effect only; model 2: full mediation model; model 3: partial mediation model. CFI = Comparative Fit Index. TLI = Tucker Lewis Index. RMSEA = Root Mean Square Error of Approximation. saBIC = Sample Adjusted Bayesian Index Criterion.

**Table 3:** Results for the structural models studied using the transformational leadership variable as the predictor in the overall (a), the USA (b), and the Spanish samples (c). Role overload and conflict (mediator) were included in models 2 and 3.

	(a) Overall sample			(b) U.S. sample			(c) Spanish sample		
	*Response: Turnover Intention*			*Response: Turnover Intention*			*Response: Turnover Intention*		
	*Model 1*	*Model 2*	*Model 3*	*Model 1*	*Model 2*	*Model 3*	*Model 1*	*Model 2*	*Model 3*
Chi-square	413.72	395.54	390.32	340.9	327.25	327.07	299.29	294.07	288.73
*Df*	133	133	132	133	133	132	133	133	132
*p* value	<0.001	<0.001	<0.001	<0.001	<0.001	<0.001	<0.001	<0.001	<0.001
CFI	0.95	0.95	0.95	0.91	0.92	0.92	0.94	0.95	0.95
TLI	0.94	0.94	0.94	0.90	0.90	0.90	0.94	0.94	0.94
RMSEA (90% CI)	0.07 (0.06–0.08)	0.07 (0.06–0.08)	0.07 (0.06–0.08)	0.09 (0.08–0.1)	0.09 (0.07–0.1)	0.09 (0.07–0.1)	0.08 (0.06–0.09)	0.07 (0.06–0.09)	0.07 (0.06–0.08)
saBIC	21553.13	21534.95	21532.61	8339.31	8325.66	8327.62	12989.05	12983.82	12980.7

	*Response: Anxiety*			*Response: Anxiety*			*Response: Anxiety*		
	*Model 1*	*Model 2*	*Model 3*	*Model 1*	*Model 2*	*Model 3*	*Model 1*	*Model 2*	*Model 3*
Chi-square	522.54	449.28	448.96	423.27	405.40	405.17	372.33	326.69	325.37
*Df*	150	150	149	150	150	149	150	150	149
*p* value	<0.001	<0.001	<0.001	<0.001	<0.001	<0.001	<0.001	<0.001	<0.001
CFI	0.93	0.94	0.94	0.89	0.9	0.9	0.92	0.94	0.94
TLI	0.92	0.93	0.93	0.88	0.89	0.88	0.91	0.93	0.93
RMSEA (90% CI)	0.08 (0.07–0.08)	0.07 (0.06–0.08)	0.07 (0.06–0.08)	0.09 (0.08–0.11)	0.09 (0.08–0.09)	0.09 (0.08–0.09)	0.08 (0.07–0.09)	0.07 (0.06–0.08)	0.07 (0.06–0.08)
SABIC	23455.08	23381.82	23384.37	8833.85	8815.97	8817.88	14332.1	14286.46	14287.36

*Note.* Model 1: direct effect only; model 2: full mediation model; model 3: partial mediation model. CFI = Comparative Fit Index. TLI = Tucker Lewis Index. RMSEA = Root Mean Square Error of Approximation. saBIC = Sample Adjusted Bayesian Index Criterion.

## Data Availability

Data are available upon request. Please contact the authors by e-mail.
